# Large-scale prevention trials could provide stronger evidence for decision-makers: Opportunities to design and report with a focus on the benefit–harm balance

**DOI:** 10.1177/17407745211068549

**Published:** 2022-02-13

**Authors:** Hélène E Aschmann, John J McNeil, Milo A Puhan

**Affiliations:** 1 Epidemiology, Biostatistics and Prevention Institute, Department of Epidemiology, University of Zurich, Zurich, Switzerland; 2 Department of Epidemiology & Biostatistics, University of California San Francisco, San Francisco, CA, USA; 3 School of Public Health and Preventive Medicine, Faculty of Medicine, Nursing and Health Sciences, Monash University, Melbourne, VIC, Australia; 4 Department of Epidemiology, Johns Hopkins Bloomberg School of Public Health, Baltimore, MD, USA

Randomized controlled trials (RCTs) are typically designed for a single specific primary outcome, and their reporting focuses on this outcome. Large-scale trials, prevention studies in particular, could yield more valuable and relevant evidence by focusing on the benefit–harm balance. These results would be more helpful for informing guideline development, health policy and individual treatment decisions. Currently, RCTs are often inconclusive and sometimes misleading with respect to the balance of benefits (i.e. efficacy outcomes) and harms (e.g. side effects or treatment burden) of interventions, which is ultimately of interest to decision-makers (such as patients, healthcare providers or guideline developers). Little of the recent progress in methods to evaluate the balance of benefits and harms of interventions has been applied to improve the design and reporting of RCTs. There is still a lack of consensus on how to design RCTs specifically to address the benefit–harm balance and contribute better evidence for decision-making. We propose three fundamental changes to the current practice to maximize how much RCTs, in particular prevention trials, can increase the certainty when estimating the benefit–harm balance of interventions.

First, we call for defining the benefit–harm balance as the primary aim of large-scale prevention RCTs. Since the primary outcome sets the focus in how the results should be interpreted,^1^ the common practice of selecting single benefit outcomes as primary outcomes generally emphasizes benefits and reduces the RCTs’ value for interpreting the benefit–harm balance. For example, after an expert panel decided that a top priority question was whether a lower systolic blood pressure target reduces cardiovascular events more than a standard target in people with hypertension and without diabetes,^2^ cardiovascular benefits were designated as the primary aim. Accordingly, the Systolic Blood Pressure Intervention Trial (SPRINT) was designed as an efficacy trial and was stopped early when the primary outcome, cardiovascular events, was significantly reduced with the lower target.^2^ As a large, high-quality, definitive study with a unique comparison of blood pressure targets, SPRINT was well-positioned to directly inform guidelines and trigger guideline updates. But a debate arose around the clinical relevance and a potentially inappropriate focus on benefits. Finally, guideline developers disagreed whether benefits outweigh increased rates of adverse events like acute kidney injury or increased treatment burden, ultimately resulting in two conflicting US guidelines.^3,4^

Specifying a primary outcome that more directly informs the benefit–harm balance reduces the risk of multiple testing and misleading interpretation of study results. For example, the Aspirin in Reducing Events in the Elderly (ASPREE) trial used disability-free survival as a primary outcome.^5^ Rather than just showing a debatable benefit–harm balance of less myocardial infarctions at the cost of more gastrointestinal bleeds, ASPREE demonstrated that aspirin did not prolong disability-free survival. If such composite outcomes are the primary outcome, they need to fulfill fundamental criteria to be interpretable.^6^ They should also be highly relevant to the target population, such as disability-free survival for the elderly.

Second, we call for large-scale RCTs to have sufficient statistical power to explicitly measure clinically relevant differences in the benefit–harm balance. Current guidance proposes that studies should be powered for multiple patient-important outcomes to ensure that benefit–harm balance can be assessed,^1^ but is otherwise non-specific. It is typically neither feasible nor necessary to power the study for *all* patient-important outcomes, or (often) for all outcomes in a core outcome set. Instead, we propose that sample size calculations should aim at estimating a metric for the benefit–harm balance (e.g. disability-free survival, survival with good function, or probability of net benefit) as precisely as needed for decision-making, based on expected treatment effects on both benefits and harms and taking into account both baseline risks and the relative importance of outcomes.

Benefit–harm metrics are useful to compare multiple outcomes on a common scale and to model the impact of additional evidence on the benefit–harm balance.^7–9^ To inform RCT design, such benefit–harm metrics should be sensitive to key patient-important benefits and harms and be responsive to the intervention. The duration of RCTs should reflect time-frames relevant to stakeholders in which both benefits and harms occur, as treatments or preventive and screening interventions may cause some benefits or harms earlier than others. Powering for the benefit–harm balance will require a larger sample size than powering for the composite of all benefit outcomes, as is often done. However, if there is more than one benefit outcome, compared to powering for a single benefit outcome only, the sample size could both decrease or increase when powering for the benefit–harm balance instead. By powering RCTs for a benefit–harm metric, RCTs will generate more valid and precise evidence for the benefit–harm balance. This will also avoid stopping large supposedly definitive RCTs like SPRINT based on benefit alone, and instead allow formulating explicit stopping rules for net benefit, net harm or futility.

Third, we advocate for nested patient preference surveys, as preference surveys could strongly guide the interpretation of results and impact guideline development and policymaking. Patient preferences can inform the choice of the outcomes and their relative importance. Decision-makers need to consider the patients’ perspective to balance benefits against harms and determine clinical relevance. In the absence of such evidence, guidelines can contradict each other, as in the blood pressure target example above. Although some evidence on patient preferences may be available, it is unlikely that any preference survey designed and performed independently of an RCT will include all outcomes (or health states) of interest and that outcome descriptions in the survey match outcome definitions of the RCT. Furthermore, respondents of surveys performed separately may not represent the trial or target population well.

Moreover, for large definitive RCTs like SPRINT, it is feasible to perform sufficiently large nested preference surveys to additionally determine the impact of variation in preferences between individuals. In contrast, guideline panels do not typically have the resources to perform large, applicable preference surveys. In a recent research project, we performed our own preference survey in patients with hypertension using best-worst scaling, a ranking exercise where the respondent repeatedly chooses the best and the worst outcome in different combinations.^10^ We could show that there is large variation in preferences between individuals, and that individual preferences can shift the benefit–harm balance of blood pressure targets.^11^ Guideline developers highly valued this result and suggested shared decision-making would be appropriate.^12^ We also found that patient preference surveys are difficult to perform for guideline developers: funding may frequently be lacking, and contacting the right patients may only be feasible through collaboration with care delivery groups with learning health systems, which can identify and contact a representative sample of members of the target population.^12^ Therefore, nested surveys in RCTs will likely provide the most applicable, valid and precise evidence on preferences to many guideline panels.

In summary, we propose a major change in the culture for large-scale prevention RCTs to primarily aim to increase the certainty in the benefit–harm balance rather than in single benefit outcomes. In particular, definitive RCTs should aim to establish net benefit, net harm or equivalence. This approach requires thorough stakeholder engagement, in particular to ensure all patient-important outcomes are considered.^12^ Furthermore, the ethics committee and data and safety monitoring boards would need to accept the benefit–harm metric as valid to determine equipoise. Given the disproportionate focus of RCTs on benefits and often lacking evidence on harms, it is not surprising that guideline developers frequently come to conflicting conclusions although often based on the same RCTs. Since RCTs are the main source of information for guideline development, policies and ultimately individual decision-making, a design and reporting that focuses more on the benefit–harm balance will help RCTs to provide high-quality, actionable evidence.
